# Formation of metallic cation-oxygen network for anomalous thermal expansion coefficients in binary phosphate glass

**DOI:** 10.1038/ncomms15449

**Published:** 2017-05-31

**Authors:** Yohei Onodera, Shinji Kohara, Hirokazu Masai, Akitoshi Koreeda, Shun Okamura, Takahiro Ohkubo

**Affiliations:** 1 Research Reactor Institute, Kyoto University 2-1010 Asashiro-nishi, Kumatori-cho, Sennan-gun 590-0494, Japan; 2 Light/Quantum Beam Field, Research Center for Advanced Measurement and Characterization, National Institute for Materials Science (NIMS), 1-1-1 Kouto, Sayo-cho, Sayo-gun 679-5148, Japan; 3 Information Integrated Materials Design Field, Center for Materials Research by Information Integration, NIMS, 1-2-1 Sengen, Tsukuba 305-0047, Japan; 4 PRESTO, Japan Science and Technology Agency, 7 Gobancho, Chiyoda-ku, Tokyo 102-0076, Japan; 5 Research and Utilization Division, Japan Synchrotron Radiation Research Institute/SPring-8, 1-1-1 Kouto, Sayo-cho 679-5198, Japan; 6 Institute for Chemical Research, Kyoto University, Gokasho, Uji 611-0011, Japan; 7 Department of Physical Sciences, Ritsumeikan University, 1-1-1 Nojihigashi, Kusatsu 525-8577, Japan; 8 Graduate School & Faculty of Engineering, Chiba University, 1-33, Yayoi-cho, Chiba 263-8522, Japan

## Abstract

Understanding glass structure is still challenging due to the result of disorder, although novel materials design on the basis of atomistic structure has been strongly demanded. Here we report on the atomic structures of the zinc phosphate glass determined by reverse Monte Carlo modelling based on diffraction and spectroscopic data. The zinc-rich glass exhibits the network formed by ZnO_*x*_ (averaged *x*<4) polyhedra. Although the elastic modulus, refractive index and glass transition temperature of the zinc phosphate glass monotonically increase with the amount of ZnO, we find for the first time that the thermal expansion coefficient is very sensitive to the substitution of the phosphate chain network by a network consisting of Zn-O units in zinc-rich glass. Our results imply that the control of the structure of intermediate groups may enable new functionalities in the design of oxide glass materials.

## Abstract (web summary)

Physical properties of glasses are closely related to their amorphous structures, which can form complex networks depending on the chemical constituents. Here, Onodera *et al*. unlock this relationship in a zinc phosphate glass at an atomistic level, which may shed light on new material designs.

Oxide glass prepared by melt-quenching is usually composed of network former (NWF) and network modifier (NWM) groups^1^. SiO_2_ is a typical NWF oxide that constitutes the ring structures, which are formed from SiO_4_ tetrahedra^2^. Alkali silicate glass consisting of an alkali oxide as NWM and SiO_2_ as NWF is a representative oxide glass and it is widely used in industrial applications such as photonic and electronic devices. Furthermore, structural modification of the SiO_4_ tetrahedral network in alkali silicate glasses has been extensively studied both experimentally and theoretically^3^,^4^,^5^,^6^ to clarify structure–property relations of glasses.

P_2_O_5_ is generally classified as NWF groups along with SiO_2_, B_2_O_3_ and GeO_2_ from the viewpoint of glass-forming ability. However, P_2_O_5_ differs from other NWF oxides since the P=O bond allows the delocalized electrons in phosphate glasses^7^,^8^,^9^,^10^. On the other hand, several studies have shown that the main glass network^11^,^12^ of non-conventional phosphate glass, that is, so-called invert glass, is composed of a counter metal oxide. Previous studies have suggested that the local structure of the PO_4_ unit does not always act as a NWF, and that its structure depends on the chemical composition of the glass. However, the detailed structure of the glass network has not been fully understood due to the lack of experimental information, which, in turn, is the result of disorder. Phosphate glass is often considered for practical applications because of its durability^13^, but has significant potential for application to several other uses, owing to its unique physical and structural properties^14^,^15^,^16^,^17^,^18^. Understanding the network structure of a phosphate glass system is therefore one of the most important unresolved issues facing glass science.

In contrast to the conventional NWF group, several metal oxides, which are classified as intermediate groups, can act as either NWF or NWM groups^1^, depending on the number of NWF units, that is, the glass composition. This suggests that the conventional glass-forming theory cannot explain the behaviour of the intermediate group of oxide glass. Zinc oxide is classified as being part of the intermediate group. In the case of zinc phosphate (ZP), monolithic bulk glass can be obtained although the P_2_O_5_ content is <33.3 mol% (that is, less than that of pyro-ZP). ZP exhibits low-melting-point properties and does not contain any hazardous cations, such as lead. Therefore, ZP glass is a promising material for use as lead-free sealing glass^19^,^20^. Recently, ZP glass has also been shown to be a good host for emitting centres^21^,^22^,^23^,^24^ and is, therefore, a potential candidate for next-generation optical materials. In fact, these types of glass exhibit high transparency in the ultraviolet region; for example, the 60ZnO–40P_2_O_5_ glass has an optical absorption edge of over 6 eV, which stems from the O–Zn charge transfer transition^25^. The bandgap of ZP glass is, however, much wider than expected especially when that (at 3.4 eV) of the wurtzite ZnO crystal with Zn–O four coordination is considered^26^. The four-coordinated state of Zn is believed to result from its having a similar coordination to that of the ZnO crystal. The structure of ZP glass, particularly that of 60ZnO–40P_2_O_5_, has been extensively analysed using X-ray photoelectron spectroscopy^27^,^28^, ^31^P nuclear magnetic resonance (NMR)^29^,^30^,^31^, X-ray or neutron diffraction^32^,^33^,^34^, high-performance liquid chromatography analysis^35^, Raman and infrared spectroscopy^36^, as well as molecular dynamics simulation^37^. Several groups have also performed structural analyses using neutron and X-ray diffraction with the aid of reverse Monte Carlo (RMC) modelling^34^,^38^,^39^. Those studies revealed the zinc in the ZP glass to be in the four-coordinated state.

However, previous investigations have shown that metal oxide has a small oxygen coordination in glass^40^,^41^,^42^,^43^, since the rigid glass network enables the metastable species of metal oxide to be sustained in the glass matrix. Therefore, the modelling of a reliable atomic arrangement in glass based on metal–cation-specific experimental data and conventional diffraction data is essential. As such, in this article, we report on the reliable atomic configuration of ZP glass from the viewpoint of three-dimensional (3D) network linkage, that is, the connectivity of each oxide. We use a combination of ^31^P magic angle spinning (MAS) NMR, Zn *K*-edge extended X-ray absorption fine structure (EXAFS), as well as X-ray and neutron diffraction data^38^ to determine the dependence of this connectivity on the chemical composition and on the zinc coordination. Moreover, we discuss the relationship between several properties and glass structure and find that the thermal expansion coefficient is sensitive to the substitution of the phosphate chain network by a network consisting of Zn–O units in zinc-rich glass.

## Results

### Sample characterization and physicochemical properties

First of all, it is ensured that nanocrystallites are not precipitated in transparent *x*ZnO-(100-*x*)P_2_O_5_ (*x*ZP) glass prepared by the melt-quench method. The chemical compositions of ZP glasses were determined by using inductively coupled plasma-atomic emission spectrometry analysis (Supplementary Fig. 1 and Supplementary Table 1). It is confirmed that the compositions of our samples are precise. Supplementary Table 2 summarizes the glass transition temperature *T*_g_, density, molar volume, refractive indices and the longitudinal sound velocity *V*_L_ of ZP glass. As the table indicates, the *T*_g_, density and refractive index increase with the amount of ZnO. Supplementary Fig. 2 shows that the longitudinal modulus *c*_11_ also increases with the amount of ZnO, while the molar volume decreases.

**Figure 1:**
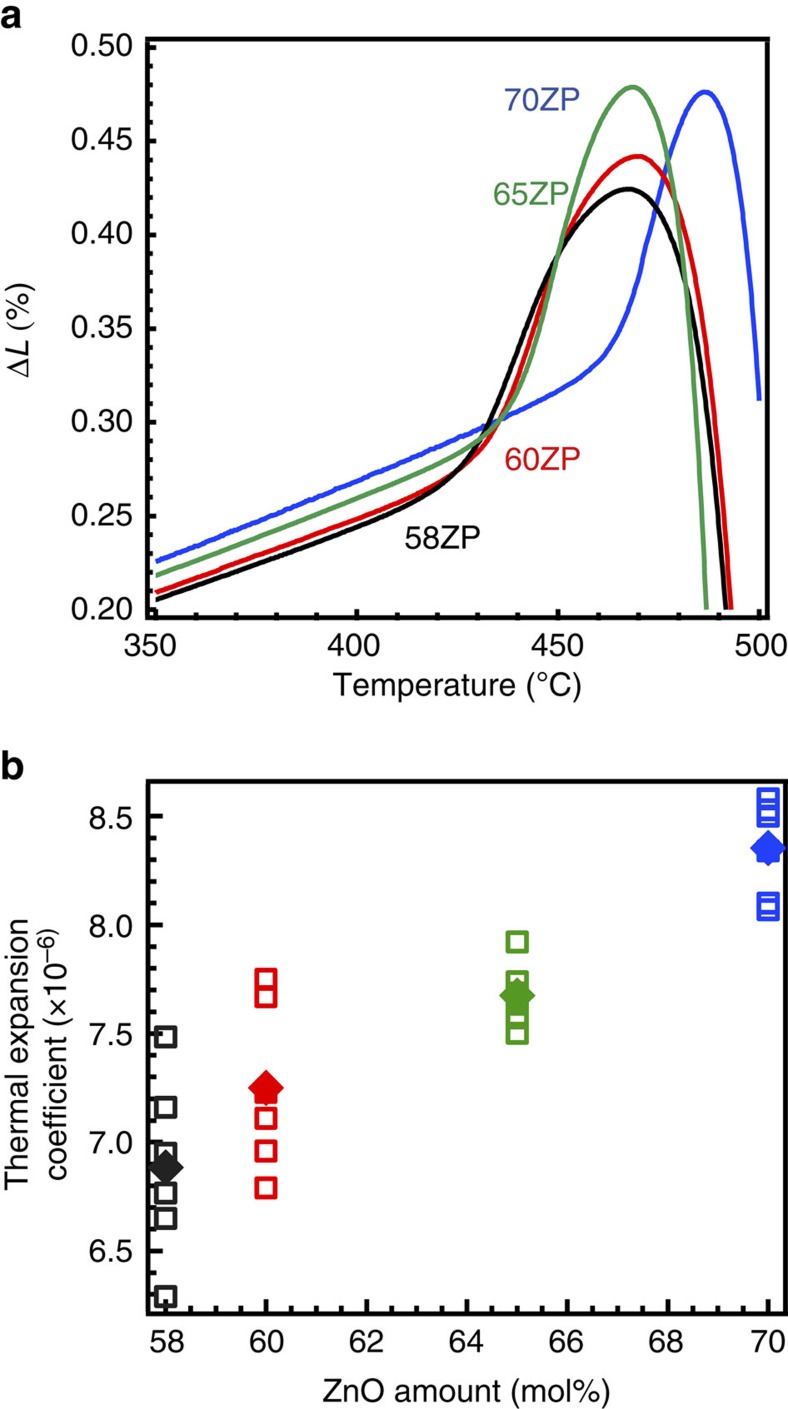
Linear thermal expansion coefficients of the ZP glass. (**a**) Thermal expansion curves of 58ZP, 60ZP, 65ZP and 70ZP glasses. (**b**) Thermal expansion coefficients below the *T*_g_ as a function of ZnO. The 70ZP glass exhibits higher *T*_g_ as well as higher thermal expansion coefficient below and above the *T*_g_, compared to those of 60ZP glass. Closed squares indicate each mean value.

Figure 1a shows the thermal expansion curves for 58ZP, 60ZP, 65ZP and 70ZP glass. The bending points of the curves correspond to the *T*_g_ listed in Supplementary Table 2. Figure 1b shows linear thermal expansion coefficients of these glasses as a function of ZnO amount. The thermal expansion coefficients increase with increasing ZnO fraction. If the glass network is similar, that is, if the glass has the same NWF, the lower *T*_g_ glass generally exhibits a higher thermal expansion coefficient^44^,^45^. However, the obtained results show that the higher *T*_g_ glass exhibits a higher thermal expansion coefficient. The anomaly of thermal expansion coefficients suggests that the glass networks might be changed, although the *T*_g_ and *c*_11_ values changed linearly depending on the chemical composition (Supplementary Table 2).

### NMR spectroscopy and EXAFS

**Figure 2:**
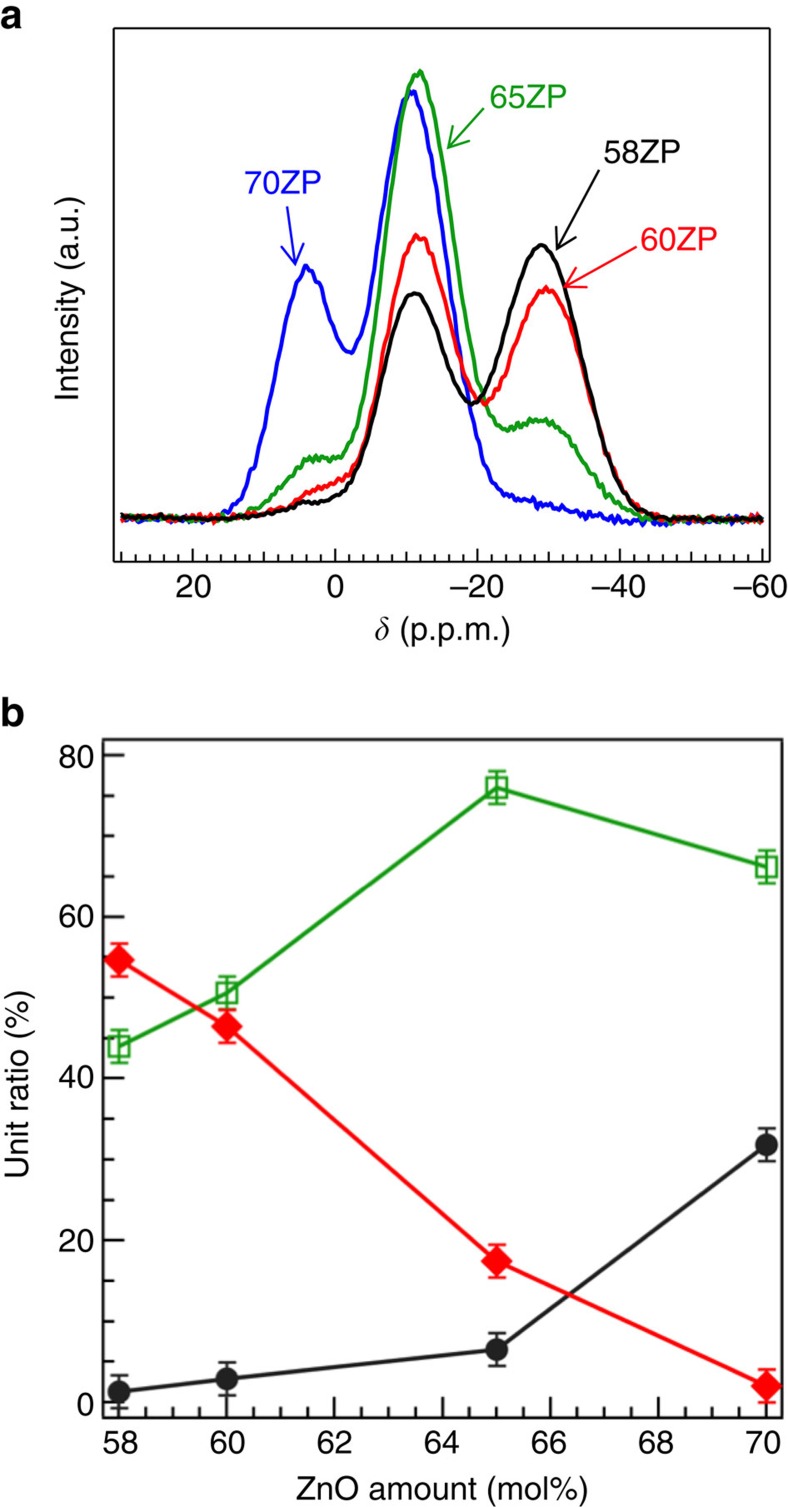
^31^P MAS NMR spectra of the ZP glass. (**a**) ^31^P MAS NMR spectra and (**b**) Q^*n*^ distribution. The peak areas are normalized using the amount of P_2_O_5_. The error bars in **b** represent s.d. of each peak area obtained from deconvolution of ^31^P MAS NMR spectra.

NMR spectroscopic measurements were used to obtain the information on the local structure in glasses. The ^31^P MAS NMR spectra of *x*ZP glass (Fig. 2a) exhibit peaks that correspond to the Q^0^ (3 p.p.m.), Q^1^ (−11 p.p.m.) and Q^2^ (−30 p.p.m.) units, which are commonly observed in ZP glass^29^,^30^,^31^,^46^. However, peaks corresponding to the Q^3^ unit are absent from the spectra. The ratio of Q^*n*^ units, as calculated from the area of each peak, shows (Fig. 2b) that the number of Q^2^ chains decreases significantly as the amount of ZnO increases. The results of our NMR are in line with the results of high-performance liquid chromatography analysis^35^, indicating that the PO_4_ units no longer form a glass network, especially for 70ZP glass, as was recently suggested by Hoppe *et al*.^39^.

**Figure 3:**
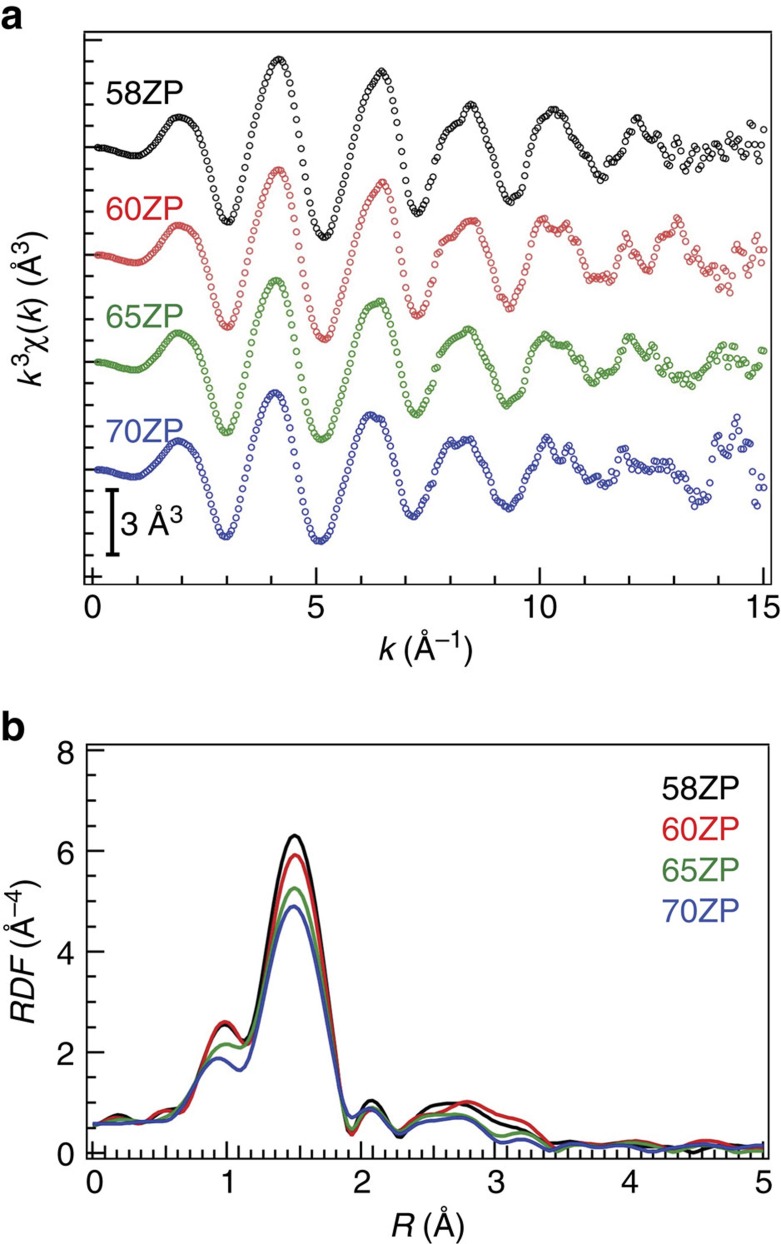
EXAFS analysis of the ZP glass. (**a**) EXAFS spectra *k*^3^*χ*(*k*) of the ZnO–P_2_O_5_ glass and (**b**) |FT(*R*)| of the spectra.

We also determined the Zn–O coordination number and subsequently the local oxygen coordination of zinc. The EXAFS spectra, *k*^3^*χ*(*k*), of the different types of ZP glass are shown in Fig. 3a. As the figure shows, the oscillation and the period of the oscillation are slightly attenuated at high *k* values and shift towards the low *k* region as the amount of ZnO increases. The Fourier-transformed (FT) EXAFS spectra of the *x*ZP glass, shown in Fig. 3b, exhibit one prominent peak, that is, the peak corresponding to the first coordination of the glass. Supplementary Table 3 summarizes the atomic distance and Debye–Waller factor, coordination number, *N*_ZnO_, of the Zn–O in the glass. We estimated the values by fitting the first coordination shell in the EXAFS spectra and obtained an atomic distance and *N*_ZnO_ of ∼1.96–1.97 Å and <4, respectively. This small oxygen coordination is different from those obtained from X-ray and neutron diffraction with the aid of RMC modelling^34^,^38^,^39^.

### Structure modelling

**Figure 4:**
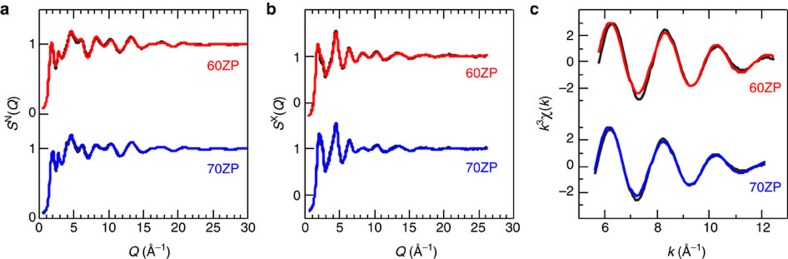
Comparison between neutron/synchrotron X-ray data and RMC model for the ZP glass. (**a**) Neutron total structure factor *S*^N^(*Q*) (**b**) X-ray total structure factor *S*^X^(*Q*) and (**c**) EXAFS *k*^3^*χ*(*k*). The EXAFS *k*^3^*χ*(*k*) were obtained by back Fourier transformation of |FT(*R*)| of the first correlation peak. Black curve, experimental data; coloured curve, RMC model.

We performed RMC modelling using X-ray diffraction, neutron diffraction^38^ and EXAFS data with several coordination number constraints to identify several chemical coordination states around phosphorus, based on the results of ^31^P MAS NMR, to reveal the behaviour of the oxygen coordination around zinc. Figure 4 shows that there is good agreement between the RMC-modelled and experimentally determined X-ray and neutron structure factors, *S*(*Q*) and EXAFS *k*^3^*χ*(*k*), of the 60ZP and the 70ZP glasses. The partial structure factors, *S*_*ij*_(*Q*), calculated from the RMC models are shown in Supplementary Fig. 3. The 60ZP and the 70ZP glasses have almost identical *S*_PO_(*Q*), *S*_OO_(*Q*) and *S*_ZnO_(*Q*), indicating that the different types of ZP glass have the same short-range structure. This similarity can also be clearly observed in the partial-pair correlation functions, *g*_*ij*_(*r*), shown in Supplementary Fig. 4. On the other hand, the *S*_PP_(*Q*), *S*_PZn_(*Q*) and *S*_ZnZn_(*Q*) differ significantly, indicating the substantial difference between the connectivity of the short-range structural units, PO_4_ tetrahedra and Zn–O polyhedra in the 60ZP and the 70ZP glasses. This behaviour is consistent with the NMR data, which reveal a significant reduction of Q^2^ species in the 70ZP glass. In addition, *N*_ZnO_ of 3.6 and 3.8 were calculated at atomic distances of up to 2.4 Å for 60ZP and 70ZP glasses, respectively. The Zn–O coordination numbers obtained from RMC modelling agree with the data determined experimentally by EXAFS, which is very sensitive to the Zn–O coordination, but are smaller than some of the values reported in previous studies^34^,^37^,^38^. To confirm the reduced Zn–O coordination in the glass, the FT EXAFS spectra and neutron total correlation functions *T*(*r*) for the different types of ZP glass were compared with those of ZnO and *β*-Zn_2_P_2_O_7_ crystals. As can be seen in Supplementary Fig. 5a, the Zn–O atomic distance in FT EXAFS spectra of the ZP glass is slightly shorter than that of ZnO crystal. In addition, the *T*(*r*) of *β*-Zn_2_P_2_O_7_ crystal shown in Supplementary Fig. 5b has two Zn–O atomic distances at 2.03 Å (Zn–O(I)) and at 2.30 Å (Zn–O(II)). The *N*_ZnO_ calculated up to the first and the second coordination distances are 4 and 2, respectively. Thus, the correlation peak at 2.03 Å of *T*(*r*) for *β*-Zn_2_P_2_O_7_ crystal is assigned to tetrahedral coordinated zinc. On the other hand, the Zn–O atomic distance is shorter and the peak area in the ZP glass is smaller than those in ZnO and *β*-Zn_2_P_2_O_7_ crystal. These observations above suggest that the Zn–O coordination in ZP glass is different from that in ZnO and *β*-Zn_2_P_2_O_7_ crystal, although fourfold zinc is dominant in glasses as Walter *et al*. concluded^30^.

## Discussion

**Figure 5:**
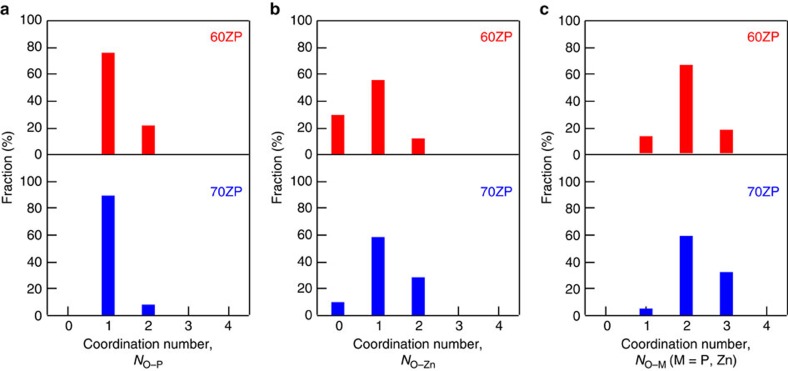
Oxygen–cation coordination number distribution in the ZP glass. The coordination number distribution of (**a**) phosphorus, (**b**) zinc (**c**) cation (phosphorus and zinc) around oxygen.

**Figure 6:**
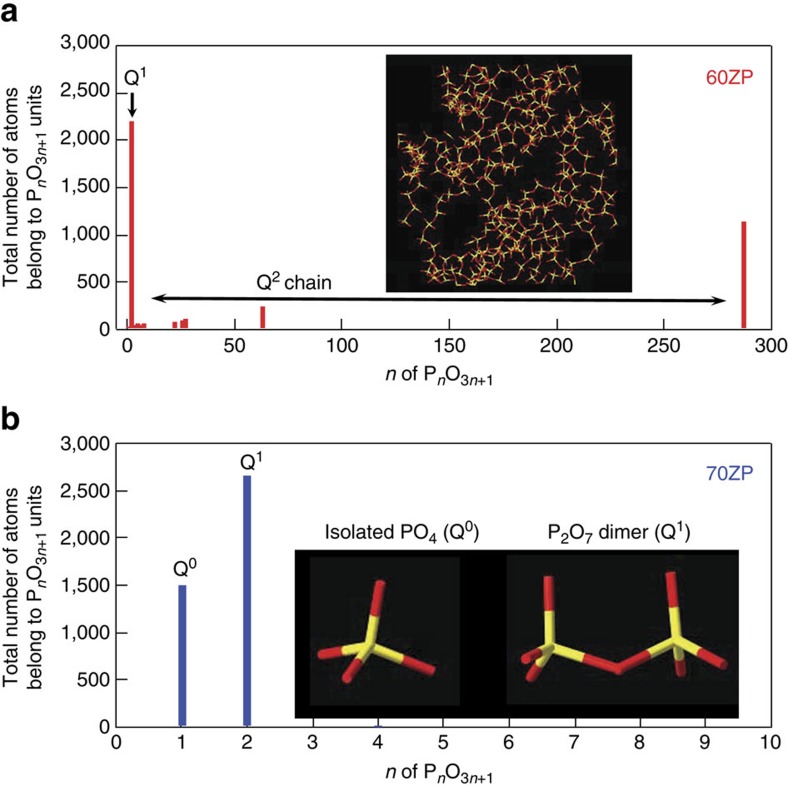
Connectivity of PO_4_ tetrahedra in the ZP glass. The size distribution of PO_4_ tetrahedral chains in **a** 60ZP glass and in **b** 70ZP glass. P and O atoms are shown in yellow and red colour, respectively.

Thus, we have succeeded in modelling atomic configurations, which reproduce the X-ray, neutron, EXAFS and NMR data. To understand network formation in the RMC models, the Q^*n*^ distribution was calculated on the basis of the atomic configurations obtained from said models. Q^0^:Q^1^:Q^2^:Q^3^:Q^4^ ratios of 0.8:49.8:49.0:0.4:0 and 33.8:65.8:0.4:0:0, obtained for the 60ZP and the 70ZP, respectively, concurred with the results of NMR shown in Fig. 2b and those reported in previous studies^29^,^30^,^31^,^35^. However, although Hoppe *et al*. reported on the structure of ZP glass derived from RMC modelling using X-ray and neutron diffraction data, we are not aware of any structure models that reproduce diffraction, EXAFS and NMR data. Accordingly, it is demonstrated that our model is reliable not at only short-range but also at intermediate range, including the connectivity of polyhedra, because our obtained Q^*n*^ distribution was calculated by analysing the –O–P–O– connectivity (not simply estimating on the basis of the coordination numbers reported in ref. 39 (see Methods for details)). The results of Q^*n*^ analysis and connectivity of atoms together with coordination numbers are listed in Supplementary Table 4. To understand the connectivity of the network consisting of PO_4_ tetrahedra and Zn–O polyhedra in detail, oxygen–cation coordination number distributions were calculated, as shown in Fig. 5. As can be seen in Fig. 5a,b, it is found that OP_2_ is more dominant than OZn_2_ in 60ZP glass, while OZn_2_ is more dominant than OP_2_ in 70ZP glass, demonstrating that the role of network formation changes from PO_4_ tetrahedra to Zn–O polyhedra between 60ZP and 70ZP, although Fig. 5c suggests that twofold oxygen is taken by an interplay between the PO_4_ tetrahedra and Zn–O polyhedra. The change in the role of the network formation from PO_4_ tetrahedra to Zn–O polyhedra between 60ZP and 70ZP can explain the behaviour of the thermal expansion coefficient. It is also found that 33% of the oxygen is coordinated by three cations (9% is P2–O–Zn1 and 24% is P1–O–Zn2) in 70ZP glass. Another important feature in oxygen–cation coordination is that the cation coordination numbers are increased in 70ZP glass. To understand such connectivity in 3D atomic configuration, the connectivity of PO_4_ and Zn–O polyhedra were analysed. Figure 6 shows the 3D linkage of the phosphate network in terms of the chain length, as calculated from the total number of atoms in each phosphate; Fig. 6a shows the fraction of Q^1^ units and Q^2^ chains of PO_4_ polyhedra in the 60ZP glass and a typical RMC-modelled Q^2^ chain. As can be seen in Fig. 6b, the 70ZP glass consists of only isolated PO_4_ tetrahedra (Q^0^) and P_2_O_7_ dimers (Q^1^). Thus, our RMC models reproduce the modification of the 3D phosphate network as observed by ^31^P MAS NMR. Similar behaviour is observed in binary silicate and aluminate glass with low glass-forming ability^40^,^41^,^42^. This observation is significant since low-melting-point phosphate glass is essential to many commercial applications.

**Figure 7:**
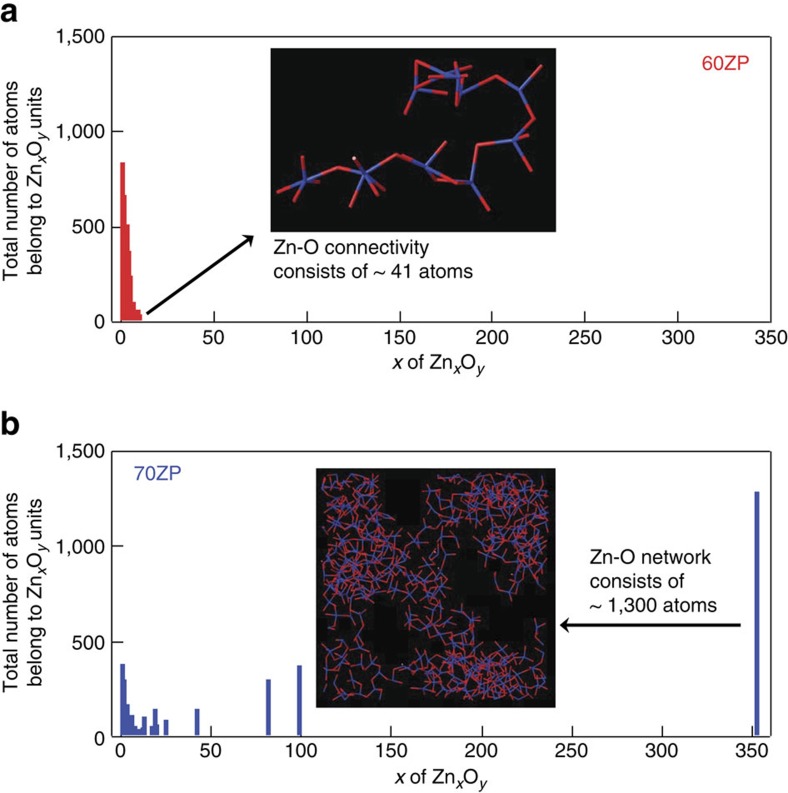
Connectivity of Zn_*x*_O_*y*_ polyhedra in the ZP glass. The size distribution of Zn_*x*_O_*y*_ polyhedral chains in **a** 60ZP glass and in **b** 70ZP glass. Zn and O atoms are shown in blue and red colour, respectively.

The probabilities of the formation of polyhedral connections between PO_4_ tetrahedra and Zn–O polyhedra were calculated (Supplementary Table 5) to elucidate the mechanism of glass formation in binary oxide glass with low amounts of NWF. Corner-sharing between PO_4_–PO_4_, PO_4_–Zn–O polyhedra and Zn–O polyhedra–Zn–O polyhedra occurred predominantly, as stipulated by Zachariasen’s rule^1^. Both types of ZP glass exhibit the same polyhedral connections, to which their similar *T*_g_ and longitudinal modulus can be attributed. Furthermore, the total number of atoms constituting the Zn_*x*_O_*y*_ units (Fig. 7) was estimated to determine the origin of the glass network in the glass. As the figure shows, the Zn_*x*_O_*y*_ units do not form a network in 60ZP glass, and the size of the fragment (consisting of up to 41 atoms, as shown in the inset of Fig. 7a) is <20 Å. In contrast, 10% of the Zn_*x*_O_*y*_ units in the 70ZP glass form networks consisting of more than 40 atoms, as manifested by their atomic configurations consisting of up to 1,300 atoms, as shown in the inset of Fig. 7b. The unusual network structure units formed by the Zn_*x*_O_*y*_ polyhedra can be attributed to the smaller oxygen coordination of the zinc compared to that of the ZnO crystal; the high glass-forming ability of the 70ZP glass stems from this small oxygen coordination. Although the Zn–O coordination number is larger in the RMC model recently reported by Hoppe *et al*.^34^, they attributed the increased oxygen coordination around the zinc to the increased rigidity of the ZP in the 75ZP and 80ZP glass. On the other hand, it is suggested from our RMC model that the small Zn–O coordination is a signature of the NWF, according to Zachariasen’s rule^1^, in our model for 70ZP glass. This suggestion is supported by the small oxygen–cation coordinations (2.06 in 60ZP and 2.29 in 70ZP) listed in Supplementary Table 4, although the oxygen–cation coordination number is increased in 70ZP glass.

In this study, several types of binary ZnO–P_2_O_5_ glass are prepared with a wide range of chemical compositions. The present findings regarding the 3D network formation of Zn_*x*_O_*y*_ units suggest that interplay between the PO_4_ tetrahedra and Zn–O polyhedra is important for tuning the physical properties of the glass. A small Zn–O coordination number might be important for improving the glass-forming ability and to increase the elastic modulus owing to this interplay. On the other hand, the addition of ZnO results in an increased oxygen–cation coordination number, which indicates an increased glass transition temperature, refractive index and packing fraction, and hence longitudinal modulus. Furthermore, we find that the thermal expansion coefficient sensitively reflects the substitution of the phosphate chain network by a network consisting of Zn_*x*_O_*y*_ units in zinc-rich glass. In other words, we succeed in revealing, for the first time, the relationship between the atomic structure of glass and its functionality by using structural modelling based on a combination of an advanced quantum beam technique and spectroscopic measurement. Since phosphate glass exhibits a low melting point, then both the phosphate and glass networks consist of counter metal oxides, which give rise to this low-melting-point behaviour. The results obtained in this study, via several structural analysis methods, are significant since they clearly reveal the fundamental properties that determine the functionality of these types of glasses and may be useful in the design of phosphate glasses for practical applications.

## Methods

### Preparation of binary ZP glass

The different types of binary ZnO–P_2_O_5_ (*x*ZP) glass, namely, 58ZnO–42P_2_O_5_ (58ZP), 60ZnO–40P_2_O_5_ (60ZP), 65ZnO–35P_2_O_5_ (65ZP) and 70ZnO–30P_2_O_5_ (70ZP), were prepared by a conventional melt-quenching method using a platinum crucible^47^. Batches consisting of ZnO (99.99%) and (NH_4_)_2_HPO_4_ (99%) were initially calcined at 800 °C for 3 h in an ambient atmosphere. The calcined solid was then melted at 1,100 °C for 30 min in an ambient atmosphere. The glass melt was quenched on a stainless plate maintained at 200 °C and then annealed at the glass transition temperature, *T*_g_, for 1 h. The samples were then mechanically polished to produce a mirror surface.

### Compositional analysis

Chemical compositions of ZP glasses were determined by using inductively coupled plasma-atomic emission spectrometry analysis using a SPECTRO BLUE (SPECTRO, Germany). The standard solutions (1,5,10 and 15 p.p.m.) of each element for the calibration curves were prepared by mixing each 1,000 p.p.m. standard solution with 0.1 M HNO_3_ solution. The calibration curves at different wavelengths of each element are shown in Supplementary Fig. 1. It was found that the calibration curves show a good linearly for both Zn and P. The concentrations of zinc and phosphorus are calculated by using averaged values of these three emission bands shown in Supplementary Fig. 1. The measured concentration values are summarized in Supplementary Table 1. The ZnO fraction *f*_ZnO_ of the glasses, that is, chemical composition of ZnO–P_2_O_5_ glasses, can be obtained from both concentration of Zn, *c*_Zn_, and that of P, *c*_P_, by using the following equation:


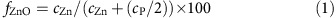


From two samples of each composition, average ZnO fraction can be calculated.

### Physicochemical analysis

The *T*_g_ was determined from thermomechanical analysis (TMA) at a heating rate of 10 °C min^−1^ and under a 1.0 g loading using a TMA 8310 (Rigaku, Japan). The linear thermal expansion coefficient of the samples, measuring around 4 mm × 4 mm × 15 mm, was also evaluated using the same equipment at a temperature range of 200–350 °C. Moreover, the local coordination state of phosphorus was determined by measuring the ^31^P MAS NMR spectra using a CMX-400 NMR spectrometer (JEOL, Japan). A frequency, spin rate and pulse delay of 161.80 MHz, 10 kHz and 5 s, respectively, were used in the measurements. The chemical shifts were estimated with respect to H_3_PO_4_ in a D_2_O solution (0 p.p.m.) and the conventional notation for phosphorus sites, Q^*n*^, was used for the analysis. The *n* value denotes the number of bridging oxygens per PO_4_ tetrahedron. Furthermore, the densities were measured by applying the Archimedes method using water at room temperature. We measured the refractive index of the samples using a prism coupler with a 473, 633, 1,319 and 1,553 nm light source (Metericon, NJ, USA); the error in the measurement was 10^−4^.

### Elastic modulus measurement

The Brillouin shifts *ν*_B_ of the different types of glass were measured using a high-resolution modification of a Sandercock Fabry–Perot system^48^. A frequency-doubled diode-pumped solid state neodymium:yttrium–aluminium–garnet laser oscillating in a single longitudinal mode at 532 nm (Oxxius SLIM-532 300 mW) was used as the excitation source. In addition to the excitation laser source, a second weak reference laser was also used to act as an independent reference signal that was used for stabilization of the Fabry–Perot. The reference laser was a small diode-pumped solid state neodymium:yttrium–vanadate (Nd:YVO_4_) laser module (Photonic Products 300-0088-01, 4 mW) oscillating in single transverse mode (TEM00), which had two to three longitudinal modes separated by 120 GHz. The longitudinal sound velocity *V*_L_, shown in Supplementary Table 2, was calculated from *V*_L_=*ν*_B_*λ*/ 2*n*_532_, where *ν*_B_, *λ* and *n*_532_ are the Brillouin shift, the wavelength of incident light (=532 nm) and the refractive index at 532 nm, respectively. The *n*_532_ values were calculated from the Cauchy relationship between the refractive indices at different wavelengths.

### EXAFS measurement

The Zn *K*-edge (9.66 keV) EXAFS spectra were measured at the BL01B1 beamline of SPring-8 synchrotron radiation facility (Hyogo, Japan). The measurements were performed using a Si (111) double-crystal monochromator in the transmission mode (Quick Scan method) at room temperature. Pellet samples for the measurements were prepared by mixing the granular sample with boron nitride. The corresponding analyses were performed by using REX2000 software (Rigaku)^49^.

### High-energy X-ray diffraction measurement

The high-energy X-ray diffraction experiment was performed at the BL04B2 beamline at the SPring-8 synchrotron radiation facility, using a two-axis diffractometer dedicated to the study of disordered materials^50^. The energy of the incident X-rays was 61.4 keV. The raw data were corrected for polarization, absorption and the background, and the contribution of Compton scattering was subtracted by using standard data analysis software^50^.

### RMC modelling

RMC modelling of the 60ZP and the 70ZP glass was performed using RMC++ code^51^. The start configurations, which contained 5,000 and 5,250 particles for the 60ZP and the 70ZP, respectively, were created using hard-sphere Monte Carlo simulations with constraints applied to avoid physically unrealistic structures. The *r-*spacing for the calculations of partial-pair correlation functions was set to be 0.05 Å. Two kinds of constraints were used: a closest atom–atom approach and connectivity. The closest atom–atom approach was chosen based on the need to avoid unreasonable spikes in the partial-pair correlation functions. The constraint on the P–O connectivity was that all of the phosphorus atoms were coordinated to four oxygen atoms for atomic distances of up to 1.7 Å. In addition, fixed neighbour constraints^52^ were applied for P–O at 1.55–1.7 Å and P=O at 1.4–1.55 Å to reproduce the Q^0^:Q^1^:Q^2^:Q^3^ ratio based on the results of the NMR in which the Q^0^:Q^1^:Q^2^:Q^3^ ratio is 0:50:50:0 for 60ZP and 33.3:66.7:0:0 for 70ZP. After the hard-sphere Monte Carlo simulations, RMC simulations containing X-ray *S*(*Q*), neutron *S*(*Q*) and *k*^3^*χ*(*k*) EXAFS data, measured at the Zn *K*-edge, were performed. The EXAFS back scattering tables were obtained from FEFF calculations^53^.

### Data availability

The authors declare that all relevant data supporting the findings of this study are available from the corresponding authors on request.

## Additional information

**How to cite this article:** Onodera, Y. *et al*. Formation of metallic cation-oxygen network for anomalous thermal expansion coefficients in binary phosphate glass. *Nat. Commun.* **8,** 15449 doi: 10.1038/ncomms15449 (2017).

**Publisher’s note:** Springer Nature remains neutral with regard to jurisdictional claims in published maps and institutional affiliations.

## Supplementary Material

Supplementary Information Supplementary Figures and Supplementary Tables
