# Supplementary material for: Formation of metallic cation-oxygen network for anomalous thermal expansion coefficients in binary phosphate glass
Source: Nat Commun. 2017 May 31;8:15449. doi: 10.1038/ncomms15449 (PMC5499210; doi:10.1038/ncomms15449)
Supplement: Supplementary Information — Supplementary Figures and Supplementary Tables [file ncomms15449-s1.pdf]

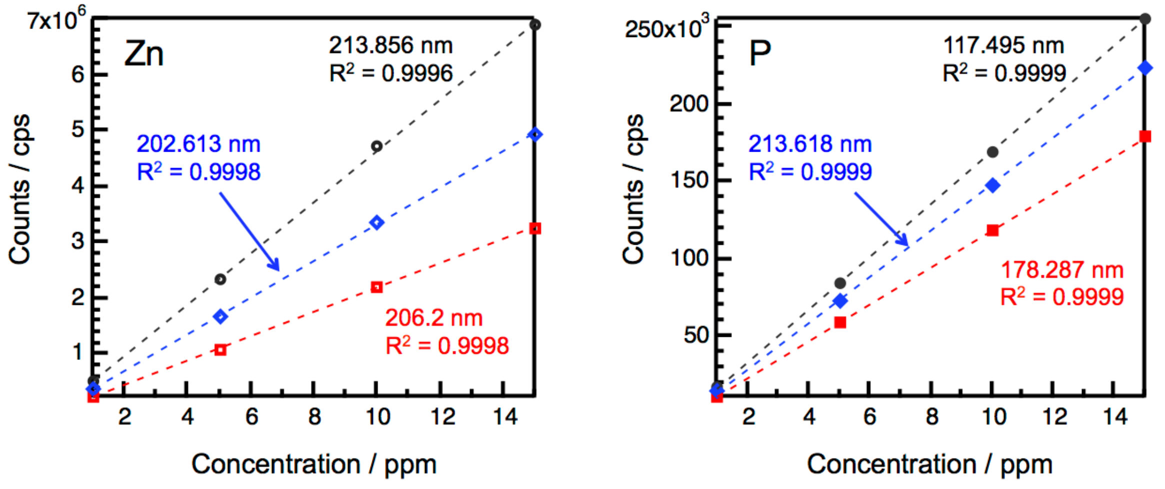

Supplementary Figure 1 | Relationship between concentration and counts of ICP-AES measurement at different monitoring wavelengths.

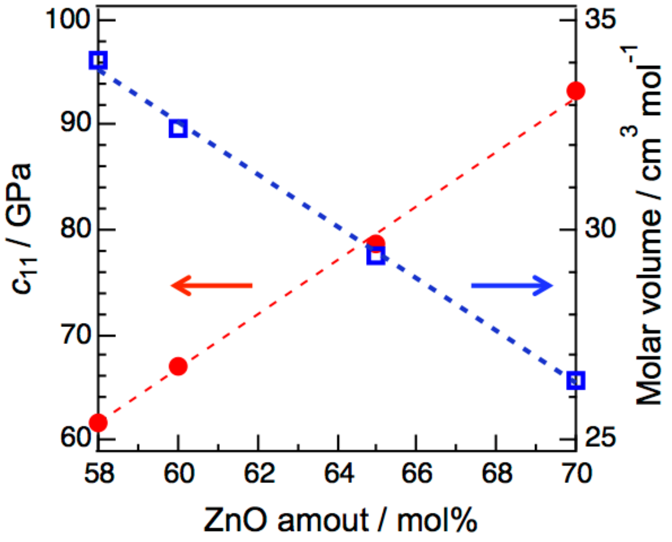

Supplementary Figure 2 | Longitudinal modulus c_{11} and molar volume for the ZP glass as a function of ZnO amount. The c_{11} value was calculated using the relation $c_{11} = \rho_m V_l^2$, where ρ_m is the density of the sample. The dashed line is a guide to the eyes.

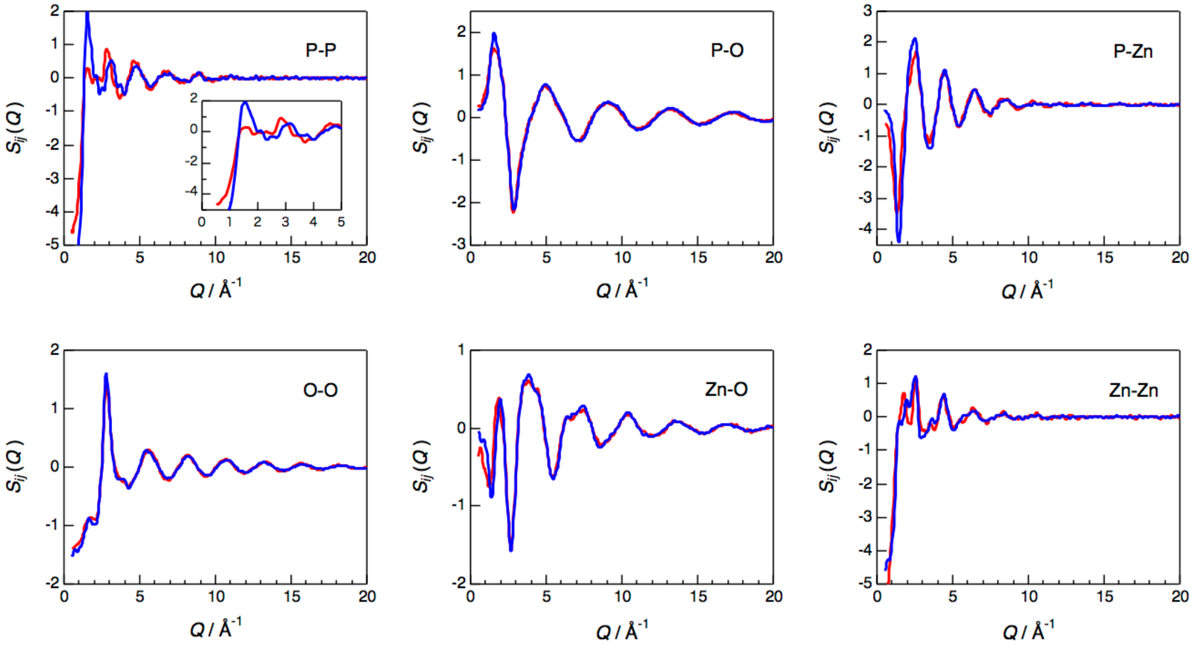

Supplementary Figure 3 | Partial structure factors $S_{ij}(Q)$ for the ZP glass obtained by RMC modelling. Red, 60ZP glass; blue, 70ZP glass.

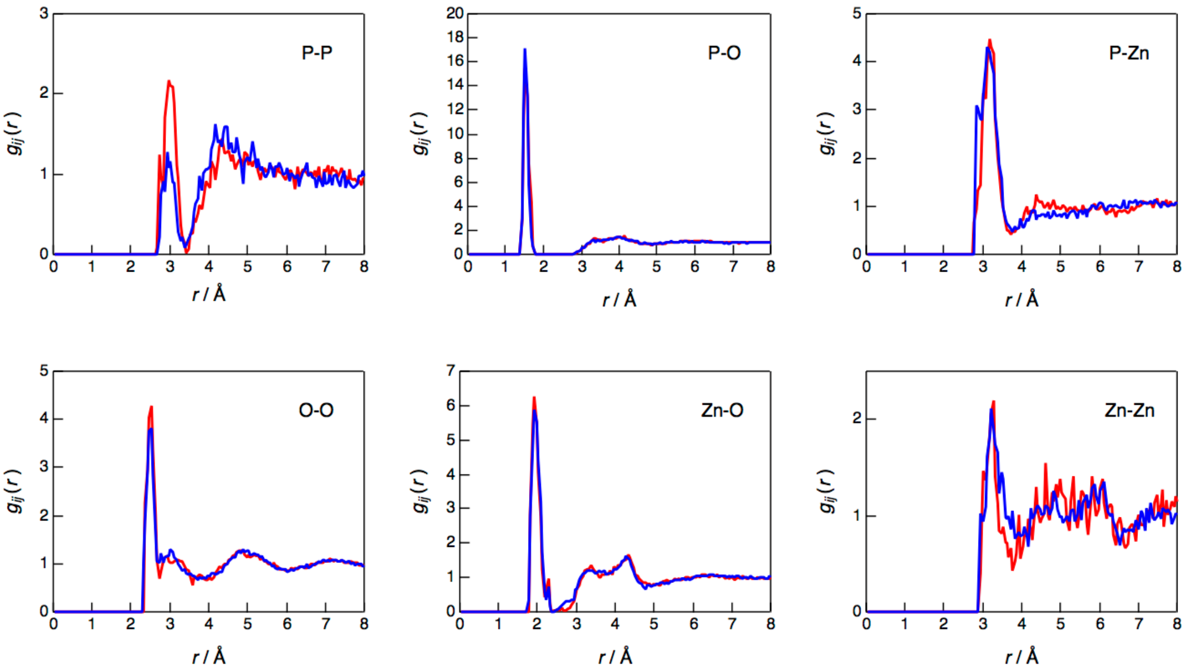

Supplementary Figure 4 | Partial pair correlation functions $g_{ij}(r)$ for the ZP glass obtained by RMC modelling. Red, 60ZP glass; blue, 70ZP glass.

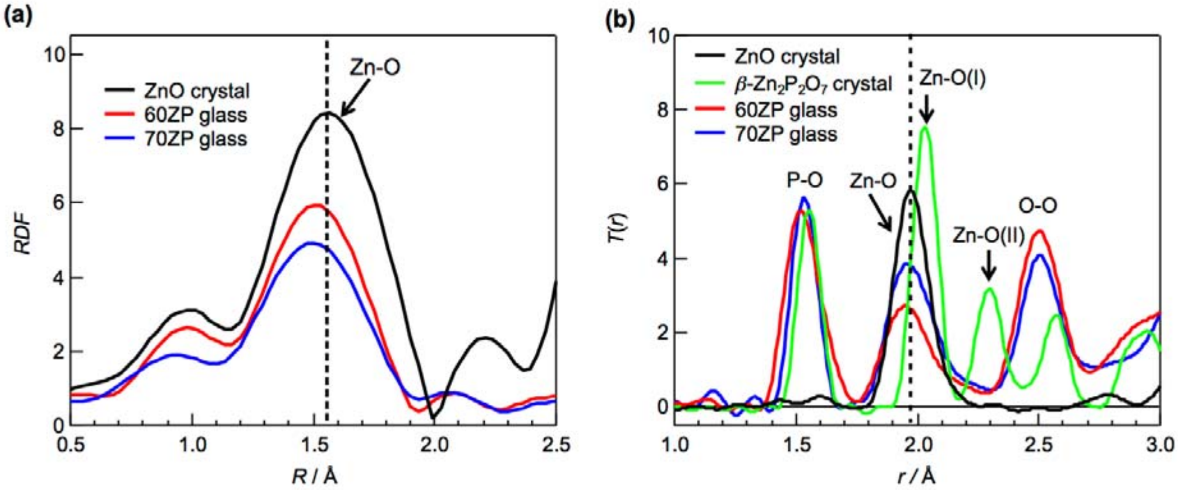

Supplementary Figure 5 | Comparison between the ZP glasses and crystals in real space. (a) Fourier transformed EXAFS spectra, *RDF,* of the ZP glass and ZnO crystal. **(b)** Neutron total correlation functions, $T(r)$, of the ZP glass, ZnO crystal and β-$Zn_2P_2O_7$ crystal. A phase shift correction of EXAFS spectra was not performed. The $T(r)$ of the β-$Zn_2P_2O_7$ crystal was calculated using PDFgui code [Farrow, C. L., Juhas, P., Liu, J. W., Bryndin, D., Bozin, E. S., Bloch, J. Proffen, Th. & Billinge, S. J. L. PDFfit2 and PDFgui: computer programs for studying nanostructure in crystals. *J. Phys.: Condens. Matter.* **19,** 335219 (2007)].

Supplementary Table 1 | Compositional analysis of the ZP glass using ICP-AES measurement. Two samples were prepared for each glass in order to obtain the average ZnO fraction.

Composition	Concentration / µmol / L		ZnO fraction / mol%	Average ZnO fraction / mol%
	c_{Zn}	c_P	f_{ZnO}	
58ZP	240	348	58.0	58.1
	231	333	58.1	
60ZP	239	315	60.3	59.9
	124	169	59.5	
65ZP	240	259	65.0	65.0
	238	256	65.0	
70ZP	221	192	69.7	69.6
	211	185	69.5	

Supplementary Table 2 | Physical parameters of the ZP glass. The sound velocities were calculated using values of the Brillouin shift ν_B, and refractive index of incident light.

Composition	T_g / °C (± 3 °C)	Density / g cm^{-3} (± 0.01 g cm^{-3})	Molar volume / cm^3 mol^{-1}	Refractive index at 532 nm (± 0.0001)	Sound velocity V_L / m s^{-1} (± 1 m s^{-1})
58ZP	424	3.14	34.0	1.5672	4,447
60ZP	431	3.26	32.4	1.5792	4,563
65ZP	437	3.49	29.4	1.6096	4,792
70ZP	461	3.77	26.4	1.6420	4,999

Supplementary Table 3 | Local coordination state of the ZP glass estimated from Zn-K edge EXAFS analysis.

Composition	Coordination number (± 0.1)	Bond length / Å (± 0.01 Å)	Debye-Waller factor / Å (± 0.01 Å)
58ZP	3.7	1.96	0.07
60ZP	3.7	1.97	0.07
65ZP	3.7	1.96	0.07
70ZP	3.6	1.97	0.08

Supplementary Table 4 | Characteristics of the RMC models of the 60ZP and 70ZP glass.
O_T, terminal oxygen; O_B, bridging oxygen; O_0, oxygen free of phosphorus.

Composition	Coordination numbers										Fraction of O atoms (in %)			Fractions of Q^n groups (in %)			
	$N_{P\text{-}O}$	$N_{O\text{-}P}$	$N_{P\text{-}P}$	$N_{O\text{-}O}$	$N_{Zn\text{-}P}$	$N_{P\text{-}Zn}$	$N_{Zn\text{-}Zn}$	$N_{Zn\text{-}O}$	$N_{O\text{-}Zn}$	$N_{O\text{-}M}$ (M=P, Zn)	O_0	O_T	O_B	Q^3	Q^2	Q^1	Q^0
60ZP	3.98	1.22	1.50	3.92	3.86	2.90	1.42	3.62	0.83	2.06	0.5	76.6	22.9	0.4	49.0	49.8	0.8
70ZP	3.98	1.09	0.71	3.88	4.04	4.71	2.39	3.78	1.20	2.29	0.4	90.5	9.1	–	0.4	65.8	33.8

Supplementary Table 5 | Probabilities (%) for polyhedral connections in the ZP glass obtained from the RMC models.

Composition	PO_4 – PO_4			PO_4 – Zn-O			Zn-O – Zn-O		
	Corner	Edge	Face	Corner	Edge	Face	Corner	Edge	Face
60ZP	100	0	0	96	4	0	85	15	0
70ZP	100	0	0	97	3	0	89	11	0
